# Supplementary material for: Behҫet’s Disease, and the Role of TNF-α and TNF-α Blockers
Source: Int J Mol Sci. 2020 Apr 27;21(9):3072. doi: 10.3390/ijms21093072 (PMC7246873; doi:10.3390/ijms21093072)
Supplement: Supplementary file 1 [file ijms-21-03072-s001.pdf]

Methods systematic review
On 10-10-2019, a PubMed search was conducted with the following strategy:

(("behcet syndrome"[MeSH Terms] OR ("behcet"[All Fields] AND "syndrome"[All Fields]) OR "behcet syndrome"[All Fields]) OR ("uveitis"[MeSH Terms] OR "uveitis"[All Fields])) AND (("infliximab"[MeSH Terms] OR "infliximab"[All Fields]) OR ("adalimumab"[MeSH Terms] OR "adalimumab"[All Fields]) OR ("etanercept"[MeSH Terms] OR "etanercept"[All Fields]) OR ("golimumab"[Supplementary Concept] OR "golimumab"[All Fields]) OR ("certolizumab pegol"[MeSH Terms] OR ("certolizumab"[All Fields] AND "pegol"[All Fields]) OR "certolizumab pegol"[All Fields] OR "certolizumab"[All Fields])) AND ((Clinical Study[ptyp] OR Comparative Study[ptyp]) AND "humans"[MeSH Terms])

In addition to this, the Cochrane database was searched, using the following terms:

(behcet syndrome OR behcet OR behcet syndrome OR uveitis OR uveitis in Title Abstract Keyword) AND (infliximab OR infliximab OR adalimumab OR adalimumab OR etanercept OR etanercept OR golimumab OR golimumab OR certolizumab pegol OR certolizumab AND pegol OR certolizumab pegol OR certolizumab) in Title Abstract Keyword - (Word variations have been searched)

The search was conducted by TH and verified by JL. Studies were first screened based on the title and abstract and, when regarded of interest, selected for inclusion based on the review of the full article. A cross-referencing search was conducted post-hoc to identify relevant studies not captured in the original search.
Quality assessment of this study was performed. Controlled clinical trials were assessed following the Cochrane tool for assessing risk of bias.[1] Risk of bias of observational studies was assessed following the Newcastle-Ottawa Quality Assessment Scale.[2]

1. Sterne JAC, Savović J, Page MJ, et al. RoB 2: A revised tool for assessing risk of bias in randomised trials. BMJ. 2019;366. doi:10.1136/bmj.l4898

2. Wells G, Shea B, O'Connell D, Peterson J, Welch V, Losos M TP. The Newcastle-Ottawa Scale (NOS) for assessing the quality of nonrandomised studies in meta-analyses. 2013.

Supplementary Table S1.

Study	Randomization process	Deviations from intended interventions	Mising outcome data	Measurement of the outcome	Selection of the reported result	Overall Bias
Melikoglu et al, 2005	Low	Low	Low	Low	Low	Low
Markomichelakis et al, 2011	High	Some concerns	Low	Low	Low	High
Zou et al, 2017	High	Some concerns	Low	Some concerns	Low	High
Martin-Varillas et al, 2018	High	Low	Low	Low	Low	High

Supplementary Table S2. Answers as given to the questions from the Newcastle Ottawa scale[2] are presented as a/b with the corresponding sentence. When contributing, a short explanation to the answer is given.

Observational Studies	Selection (Score)				Comparability (Score)	Exposure (Score)			
Cohorts studies	Representativeness of Exposed Cohort	Selection of the Non Exposed Cohort	Ascertain of exposure	No Outcome of interest at the start of the study	Control for Important Factor or Additional Factor	Assessment of Outcome (blinding)	Adequate follow up	Adequacy of follow up cohorts	Total Score
Tabbara, 2008	1 - b) Somewhat representative (Mantoux negative BD patients)	1 – a) Drawn from the same community	1 – a) Sure record (medical records)	1 – a) No outcome of interest at start	1 –a) Controls matched for age	1 – b) Medical record linkage	1 – a) Yes	1 –a) Complete follow-up of all subjects	8/9
Yamada, 2010	1 – b) Japanese BD criteria of Behçet disease Research Committee	1 – a) Drawn from the same community	1 – a) Sure record (medical records)	1 – a) No outcome of interest at start	2 –b) Controls matched for age and seks	1 – b) Medical record linkage	0 – b) No, follow up period of 6 months	1 –a) Complete follow-up of all subjects	8/9

Takeuchi, 2012	1 – b) Japanese BD criteria of Behçet disease Research Committee	1 – a) Drawn from the same community	1 – a) Sure record (medical records)	1 – a) No outcome of interest at start	1 –a) Controls matched for age	1 – b) Medical record linkage	1 – a) Yes	1 –a) Complete follow-up of all subjects	8/9
Keino, 2017	1 – b) Japanese BD criteria of Behçet disease Research Committee	1 – a) Drawn from the same community	1 – a) Sure record (medical records)	1 – a) No outcome of interest at start	1 –a) Controls matched for age	1 – b) Medical record linkage	1 – a) Yes	1 –a) Complete follow-up of all subjects	8/9
Guzelant, 2017	1 – b) BD patients with uveitis	1 – a) Drawn from the same community	1 – a) Sure record (medical records)	1 – a) No outcome of interest at start	2 –b) Controls matched for age and seks	1 – b) Medical record linkage	1 – b) No, event rate not corrected for follow up duration	1 –a) Complete follow-up of all subjects	8/9
Emmi, 2016	1 – a) Truly representative	1 – a) Drawn from the same community	1 – a) Sure record (medical records)	1 – a) No outcome of interest at start	1 –a) No adjusted analysis.	1 – b) Medical record linkage	1 – a) Yes	1 –a) Complete follow-up of all subjects	8/9
Miyagawa, 2019	1 – b) BD patients with gastro-enteric involvement	1 – a) Drawn from the same community	1 – a) Sure record (medical records)	1 – a) No outcome of interest at start	0 –a) Differences in age, sekse.	1 – b) Medical record linkage	1 – a) Yes	1 –b) Small number of subjects lost	7/9
